# Formation mechanism and characterization of porous biomass carbon for excellent performance lithium-ion batteries

**DOI:** 10.1039/c8ra02002g

**Published:** 2018-04-03

**Authors:** Yi Li, Chun Li, Hui Qi, Kaifeng Yu, Xiangji Li

**Affiliations:** Key Laboratory of Automobile Materials, Ministry of Education, College of Materials Science and Engineering, Jilin University Changchun 130025 China yukf@jlu.edu.cn xjli@jlu.edu.cn; The Second Hospital of Jilin University Changchun 130041 P. R. China

## Abstract

Porous biomass carbon derived from corn stalks was prepared *via* carbonization and activation of CaCl_2_. Combined with its microstructure, the formation mechanism and electrochemical properties were analyzed. The addition of CaCl_2_ was the key factor to form the porous structure, and the proportion of CaCl_2_ had a significant impact on the pores distribution and electrochemical properties. The resulting sample had a specific surface area of 370.6 m^2^ g^−1^ and an average pore size of 9.65 nm. The sample was circulated at 0.2C for 100 cycles, the specific discharge capacity was 783 mA h g^−1^. After 60 cycles at different rates, when the current was restored to 0.2C again, the discharge specific capacity quickly recovered. This showed that the sample had excellent rate performance and cycle stability for lithium-ion batteries.

## Abstract 2

Porous biomass carbon derived from corn stalks was prepared *via* carbonization and activation of CaCl_2_.

## Graphical abstract

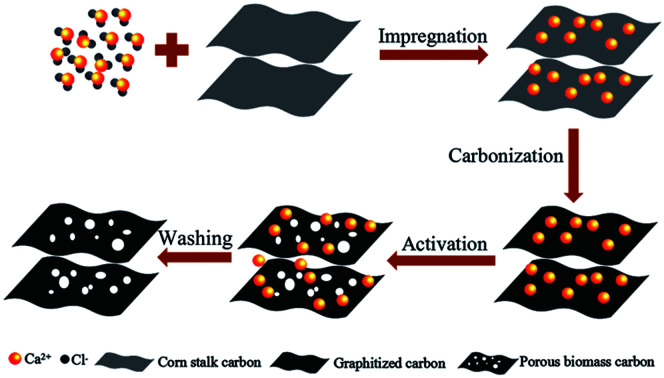

## 1. Introduction

Lithium ion batteries (LIBs) have many advantages such as high specific capacity, light weight, long cycle life and small self-discharge, so LIBs are widely used in mobile communication, notebook computers, digital cameras and other portable electronic products.^1–4^ As the main anode material, carbon has outstanding impacts on the performances of lithium ion batteries. Carbon materials mainly include graphite, soft carbon and hard carbon. Among them, graphite is the most common anode material due to its high energy density, excellent electrical conductivity, good cycle ability, and relatively low cost.^5,6^ However, graphite owns smaller layer spaces (0.335 nm) and longer diffusion distances for lithium ion, which increases the diffusion resistance for lithium ions.^7^ And charging at high rate could lead to the growth of the lithium dendrite on the graphite surface, causing short circuit inside lithium ion batteries.^8^ Therefore, a kind of new structure material needs to be found, which not only can avoid the phenomenon of lithium dendrite but has the common advantages of graphite.

The porous biomass carbon prepared by corn stalk has the advantages of adjustable pore size distribution, high specific surface area, large pore volume, good electrical conductivity and thermal conductivity.^9–11^ It could inhibit the growth of lithium dendrite and provide more transmission channels for lithium ions, which makes the prepared lithium ion batteries possibly owning higher energy density, better stability and security. The way of reusing corn stalk also can reduce the environmental pollution and realize the greening of energy.^12^ Wang^13^*et al.* synthesized a kind of porous carbon derived from rice husk, whose capacity was 137 mA h g^−1^ at 10C used as an anode in lithium ion batteries. Li^14^*et al.* prepared disordered carbon from rice husks for lithium ion batteries, whose reversible capacity was 502 mA h g^−1^ at 0.2C after 100 cycles. However, their reversible capacities were not enough high.

In order to improve the reversible specific capacity of the carbon anode material, a simple method was proposed on the basis of the traditional preparation method. The advantages are as follows: firstly, using corn stalk as carbon source could reduce environmental pollutants and reuse resources. Secondly, the size and distribution of the pores in porous carbon could be adjusted by controlling the amount of CaCl_2_. In addition, the CaCl_2_ as activator is cheap and recyclable, reducing the cost of porous carbon. More importantly, the prepared biomass carbon has excellent charge–discharge capacity and stable circulation performance.

## 2. Experiment

### 2.1 Materials preparation

The corn stalk used in this experiment was collected from the agricultural land in Jilin Province, China. The natural dried corn stalks were crushed into powers, then according to the weight ratio of 1 : 2, 1 : 2.5 and 1 : 3, corn stalk powder and CaCl_2_ were weighed and mixed uniformly. After fully impregnated, the mixture was dried at 60 °C and then was thermally carbonized in a muffle furnace at 300 °C for 3 h. After cooling, the carbonized samples were activated at 600 °C for 1 h in muffle furnace, and then cooled until room temperature. The obtained samples were firstly washed with deionized water to recycle and re-use the CaCl_2_ solution. Then the samples after washing were washed by 2 mol L^−1^ HCl solution to remove a small amount of impurities such as potassium, magnesium, calcium. Finally, the pickled samples were washed with deionized water at 80 °C until neutral, and then was dried in an oven at 60 °C. Samples prepared at mass ratios of 1 : 2, 1 : 2.5 and 1 : 3 were labeled as CSC-2, CSC-2.5 and CSC-3, respectively. The control experiment sample without CaCl_2_ treatment was named as CSC.

### 2.2 Materials characterization

The powder X-ray diffraction (XRD) measurements were carried out using a Siemens D5000 X-ray diffractometer with nickel-filtered Cu K radiation. Raman spectra was recorded on a Renishaw inVia instrument. The morphology of the samples was observed by scanning electron microscopy (JEOL JSM-7500F) and transmissions electron microscopy (JEM-2100F). The specific surface area and pore size distribution were measured using nitrogen adsorption–desorption measurements (Micromeritics, ASAP2420).

### 2.3 Electrochemical measurements

The active substance, acetylene black and polyvinyl fluoride (PVDF) were mixed at the mass ratio of 8 : 1 : 1. After grinding evenly, an appropriate amount of *N*-methyl-2-pyrrolidone (NMP) was added, then the mixture was stirred and diluted into a uniform paste. The slurry was coated on the copper foil to form an anode plate, and then was dried in a vacuum oven at 120 °C for 12 h. The anode plate was pressed into 10 mm diameter wafer as working electrode in the tablet machine. The loading quantity of active material was approximately 0.70 mg cm^−2^. Coin-type (CR2025) cells were assembled in an Ar-filled glovebox with moisture and oxygen concentrations below 1.0 ppm. The lithium foil was used as the counter electrode and the reference electrode. 1 mol L^−1^ LiPF_6_ in a 50 : 50 w/w mixture of ethylene carbonate and diethyl carbonate was used as electrolyte. The charge–discharge performance were tested between 0.02 V and 3.0 V at a 0.2C (1C = 372 mA g^−1^) rate on a LAND (CT2001A) battery test system. Cyclic voltammetry and impedance curve were performed on a CHI660C electrochemical workstation within a voltage window of 0–3.0 V at a scan rate of 0.1 mV s^−1^.

## 3. Results and discussions

**Fig. 1:**
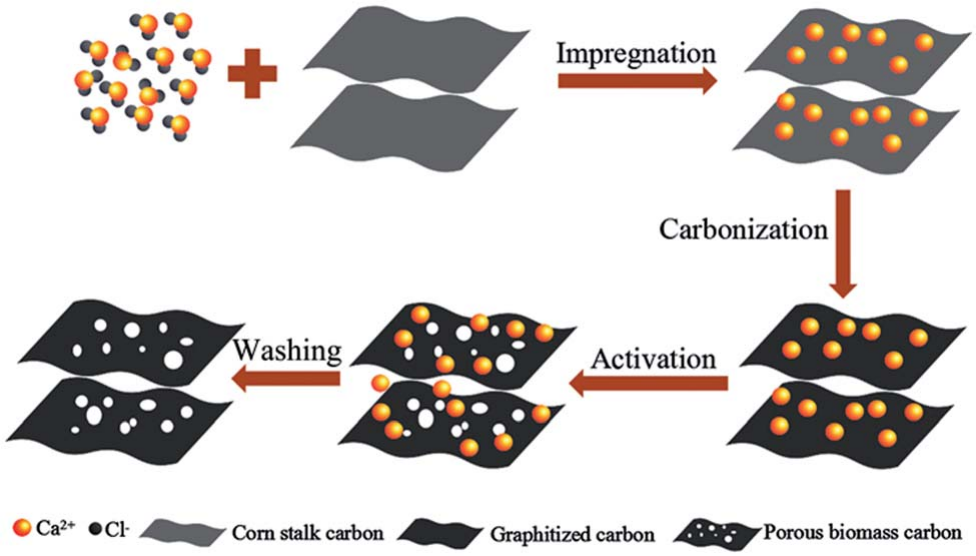
The formation mechanism of porous biomass carbon derived from corn stalk.

The formation mechanism of porous biomass carbon derived from corn stalk was shown in Fig. 1. In the process of impregnation, CaCl_2_ penetrated into the corn stalks. During the process of pyrolysis and carbonization, the dehydration of CaCl_2_ made the pyrolysis reaction easier to carry out. Under certain temperature, CaCl_2_ in corn stalks could dissolve cellulose, hemicellulose and lignin, and form pores in them. These pores became the active centers of carbon. During the carbonization process, most of the CaCl_2_ can remain in the carbon to form the skeleton, and the amorphous graphite microcrystalline structure was formed at this time. When activated by further heating, the carbon continues to decompose and rearrange on the framework to form nanoscale crystallites. This system was generally considered to be heterogeneous materials consisting of graphite grains and amorphous carbons with CaCl_2_ as the backbone. When CaCl_2_ was washed away with deionized water, a unique porous structure was formed in the end.

**Fig. 2:**
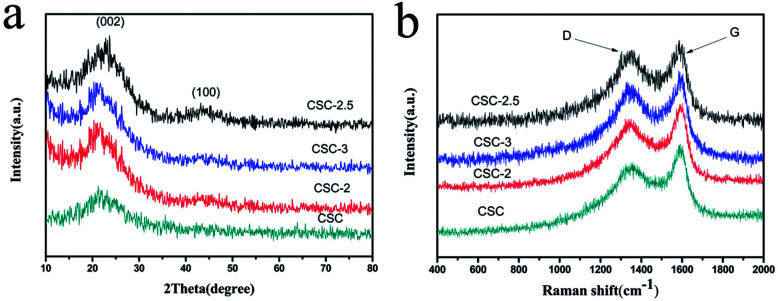
(a) XRD patterns and (b) Raman spectrum of the CSC-2.5, CSC-3, CSC-2 and CSC samples.

Fig. 2a showed the XRD patterns of the CSC-2.5, CSC-3, CSC-2 and CSC samples. It was obvious that the diffraction peaks near 22° and 43° corresponded to the (002) and (100) planes of amorphous carbon, indicating that the addition of CaCl_2_ did not change the crystal structure.^15–17^ Although the ratio of CaCl_2_ had no effect on the crystal structure, it changed the disorder degree of the samples, which was displayed in Fig. 2b*via* Raman spectrum. The G-band (∼1580 cm^−1^) was generated by the stretching motion of all sp^2^ atoms in the carbocyclic or long chain, while the D-band (∼1360 cm^−1^) was ascribed to the edges, other defects, and disordered carbon.^18,19^ The *I*_D_/*I*_G_ ratio of the CSC-2.5 sample was 0.98, significantly higher than that of the CSC (*I*_D_/*I*_G_ = 0.66), CSC-2 (*I*_D_/*I*_G_ = 0.86) and the CSC-3 (*I*_D_/*I*_G_ = 0.78) samples. This indicated the CSC-2.5 sample had a greater disorder degree, more edges and other defects, which was conducive to enhance reversible capacity and improve Li^+^ storage ability.^20^

**Fig. 3:**
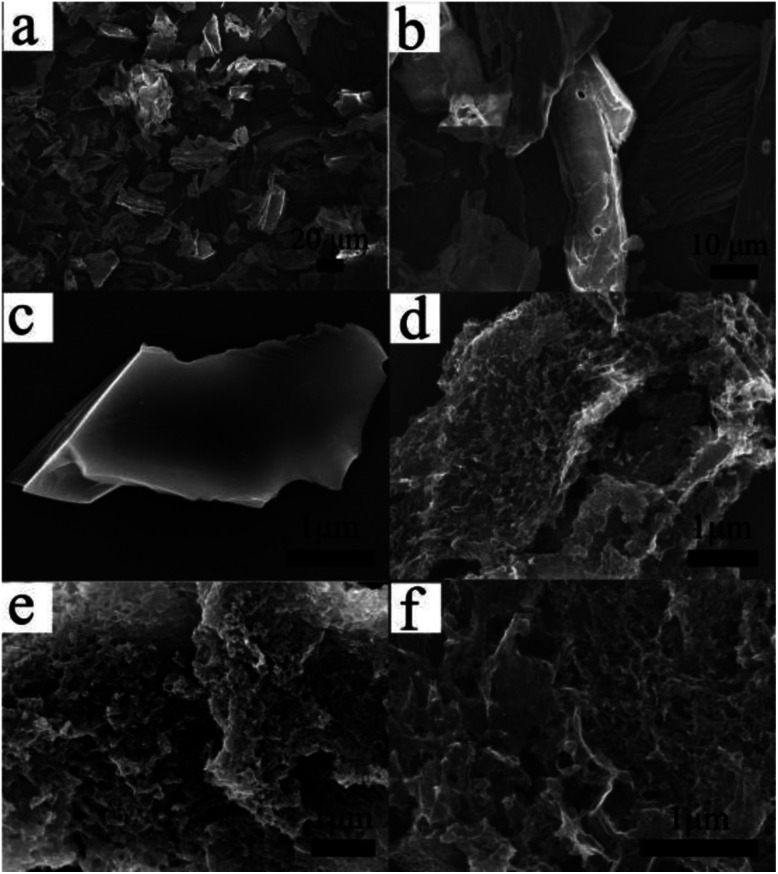
SEM images of the (a and b) corn stalks before carbonization, (c) CSC, (d) CSC-2, (e) CSC-2.5 and (f) CSC-3 samples.

The morphology structures of the samples were characterized by SEM, the results were shown in Fig. 3. It can be observed that the morphology of the corn stalks before carbonization was irregular. The surface of the CSC sample was smooth and had no irregular concave–convex structure. However, the surface of the CSC-2, CSC-2.5 and CSC-3 became significantly more rough and had different degree of pores and channels structure, indicating that CaCl_2_ was an important reason for the formation of pore structure. CSC-2 sample had fewer pores and lower degree of surface irregularities than that of CSC-2.5. The reason was that the amount of CaCl_2_ was too small to promote the formation of framework and pore structure, so a porous structure was barely formed on the surface of the sample. Due to the high proportion of CaCl_2_, some of the mesopores in CSC-3 sample were interconnected to become macropores. Compared to CSC-2 and CSC-3 samples, the CSC-2.5 sample had more pores inside the carbon layer and spaces formed by the staggering of pores and channels. This unique structure allowed the CSC-2.5 sample to have a high specific surface area and increased the contact area of reactants and the electrolyte, which could provide more sites for lithium ions to intercalate and delaminate, and was advantageous for increasing the specific capacity. After the above analysis, we can conclude that CaCl_2_ was an important reason for the formation of pore structure, and the amount of CaCl_2_ also affected the distribution of pore structure.^21^

**Fig. 4:**
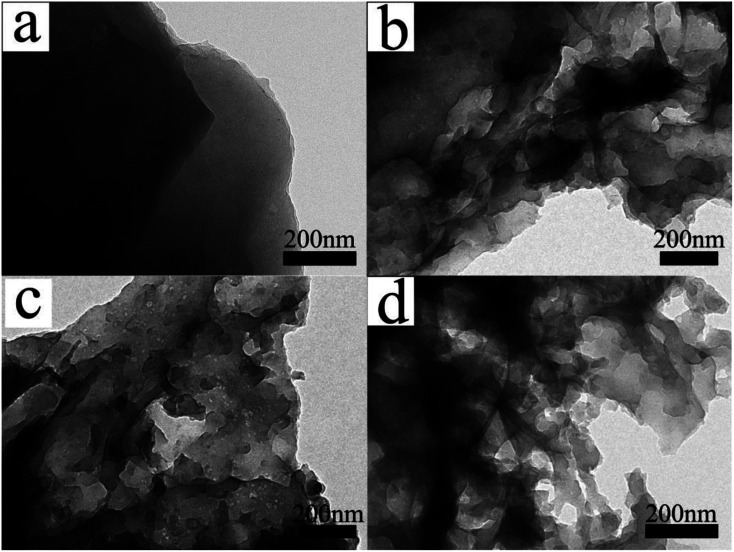
TEM images of the (a) CSC, (b) CSC-2, (c) CSC-2.5 and (d) CSC-3 samples.

The microstructures of the samples were also analyzed by transmission electron microscopy (TEM). The CSC sample in Fig. 4a image had sheet-like carbon layer structure without irregular shaped pits or mesoporous structures. In the TEM images of CSC-2, CSC-2.5, CSC-3 samples, there were disordered white dots and irregular pits distributing on the surface of the material. The white dots correspond to the mesopores in the carbon layer. The irregular pits were large pores or grooves formed by the expansion of the mesopores in the carbon layer. The mesopores and grooves deep into the carbon layer could increase the specific surface area, provide more transport channels for lithium ions and increase the specific capacity of the sample. The CSC-2 sample had less mesopores due to insufficient CaCl_2_. In CSC-3 sample, the proportion of CaCl_2_ was high, so that some mesopores expanded into large pores, which was not conducive to the storage of lithium ions. Compared with CSC-2 and CSC-3 samples, it was not difficult to find that CSC-2.5 sample had more mesoporous structures, and the distribution of grooves was more uniform, meaning larger specific surface area and better dispersibility. It could provide more active sites for the insertion and extraction of lithium ions, which was beneficial to improve the electrochemical performance. The analysis results show that CaCl_2_ was a key factor in the formation of porous sheet-like structures and could affect the pore distribution of porous carbon. When the weight ratio of corn stalk powder and CaCl_2_ was 1 : 2.5, the sample had the best dispersibility and pore distribution.

**Fig. 5:**
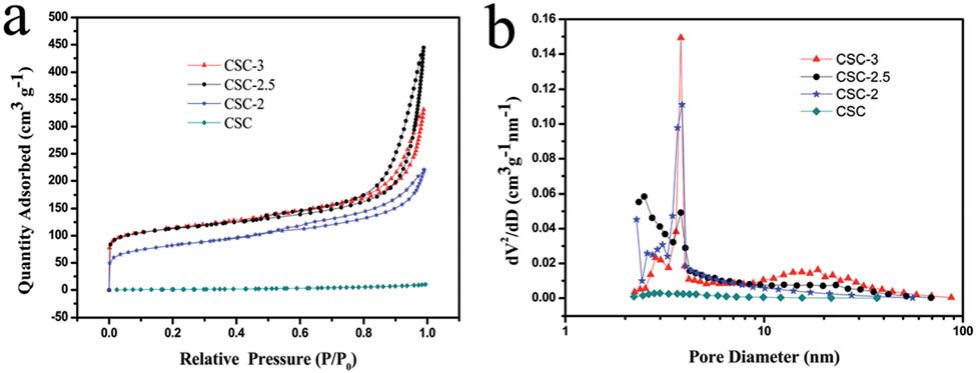
(a) Nitrogen adsorption–desorption isotherms and (b) pore sizes distribution of CSC-3, CSC-2.5, CSC-2 and CSC samples.

In order to further characterize the porous structure, the specific surface area and pore size distributions of the as-prepared samples were measured. Fig. 5a was the nitrogen adsorption–desorption isotherms of samples. The isotherm profiles of CSC sample showed that only had narrow microporous structure, and adsorption quickly reached saturation. The isotherm profiles of CSC-2, CSC-2.5, CSC-3 samples can be indexed as type IV with H_4_ hysteresis loop. A significant H_4_-type hysteresis loop was observed in the range of 0.5–1.0 relative pressure (*P*/*P*_0_), which mean the presence of mesopores (2–50 nm).^22,23^ The formation of mesoporous were mainly ascribed to the etching of CaCl_2_ during pyrolysis. The specific surface area of CSC-2, CSC-2.5, and CSC-3 samples were 267.5 m^2^ g^−1^, 370.6 m^2^ g^−1^ and 345.4 m^2^ g^−1^. The pore size distribution curve in Fig. 5b indicated the average pore sizes of CSC-2, CSC-2.5, and CSC-3 samples were 7.52 nm, 9.65 nm and 14.64 nm, respectively. The CSC-2.5 sample had larger specific surface area, which was more conducive to the insertion and extraction of lithium ions in the host material, providing a channel for the transport of lithium ions and electrons. The results of the analysis were consistent with the results of SEM and TEM.

**Fig. 6:**
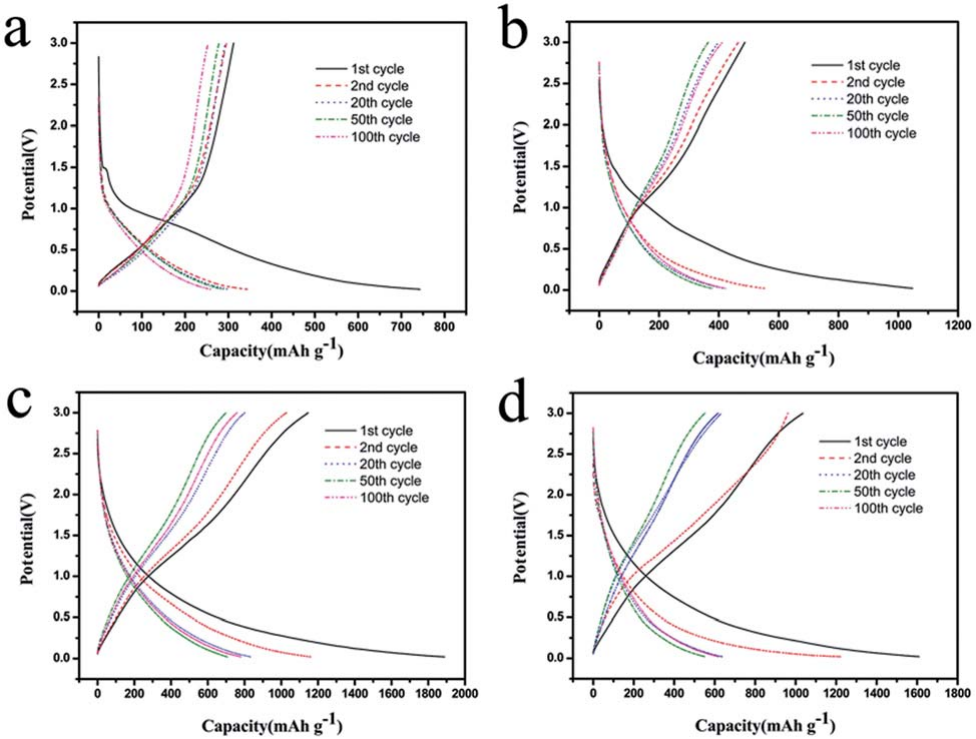
Charge–discharge curves of the (a) CSC, (b) CSC-2, (c) CSC-2.5 and (d) CSC-3 samples.

The charge–discharge curves of the samples at 0.2C were displayed in Fig. 6. In the first cycle, the discharge capacity of CSC, CSC-2, CSC-2.5, and CSC-3 samples were 742.3 mA h g^−1^, 1048.3 mA h g^−1^, 1862.1 mA h g^−1^ and 1603.2 mA h g^−1^, respectively. The high initial discharging capacity of the CSC-2, CSC-2.5, and CSC-3 sample was attributed to the unique porous structure. This structure increased the reaction area of the negative electrode material and the electrolyte, and improved the penetration ability of the electrolyte in the electrode. In addition, the large specific surface area provided more active sites for the insertion and extraction of lithium ions.^24^ The initial capacity loss could be ascribed to the conversion of the carbon electrode from their pristine form to an active lithium storage host, and the formation of solid electrolyte interface (SEI) caused by the catalytic reduction of the electrolyte components on the active electrode surface.^25^ As passivation layer, the SEI film was actually advantageous. When the SEI film reached a certain thickness, the SEI film had an insulating effect on the electrons, and prevented the electrolyte undergoing continuous decomposition on the carbon electrode. Therefore, from the beginning of the second cycle, the reversible capacity became stable, and the charge–discharge efficiency approached 100%.

**Fig. 7:**
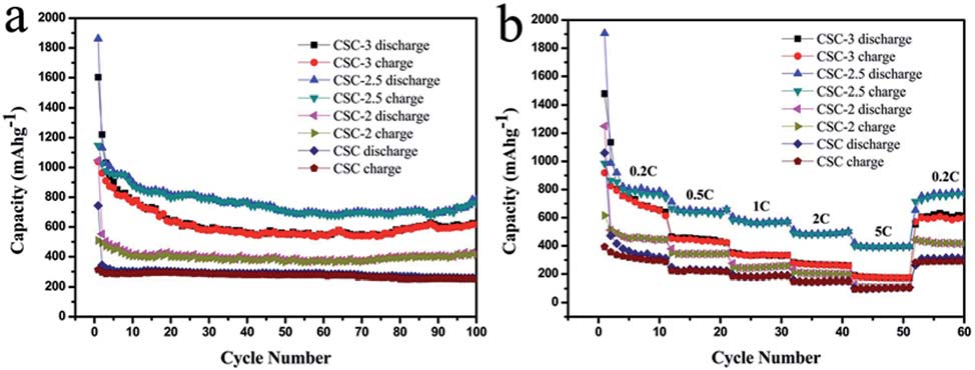
(a) Cycling performance profiles of CSC-3, CSC-2.5, CSC-2 and CSC samples at 0.2C and (b) rate performance of CSC-3, CSC-2.5, CSC-2 and CSC samples.

The cycling performance profiles of the samples at 0.2C were compared in Fig. 7a. The discharge capacities of the CSC-3, CSC-2.5, CSC-2 and CSC samples were 623.1 mA h g^−1^, 783.8 mA h g^−1^, 421.4 mA h g^−1^ and 259.2 mA h g^−1^ after 100 cycles, respectively. It was seen that the CSC sample showed lower capacities than the others, showing that porous carbon activated by CaCl_2_ had better charge and discharge performance. However, the specific capacity of CSC-2.5 sample after 100 circles was largest, indicating that larger specific surface area and more vacancies of surface were more conducive to improve the charge–discharge capacity. The first cycle coulomb efficiency of the CSC-3, CSC-2.5, CSC-2 samples were 64.8%, 60.16%, 48.5%. The reason was that SEI films were formed during lithium intercalation, which led to the loss of capacity and the decrease of coulombic efficiency. The coulombic efficiency almost remained 100% after the 5th circles, indicating that this porous carbon had excellent stability. It was not difficult to observe that the specific capacity gradually picked up from 60 circles to 100 circles. We speculated that the insertion and extraction of lithium ions might make the porous structure collapse, which led to the decrease of the volume strain energy and the increase of the charge–discharge capacity.^26,27^Fig. 7b displayed the cyclic performance of various rate at 0.2C, 0.5C, 1C, 2C, 5C, 0.2C. When the current density returned to 0.2C, the discharge capacity of all samples can be quickly restored, indicating that the material had good rate performance. It was obvious that the CSC-2.5 sample had the highest charge–discharge capacity than other samples at different rate, while the CSC sample had the lowest charge–discharge capacity among them. This was because that in the high temperature pyrolysis process, the etching of CaCl_2_ formed porous structures and surface defects, increasing specific surface area and the contact area of electrode/electrolyte interface, so the transmission distance of lithium ions was shorten and specific capacity increased. The highest specific capacity of the CSC-2.5 sample was due to the appropriate ratio of CaCl_2_.

**Fig. 8:**
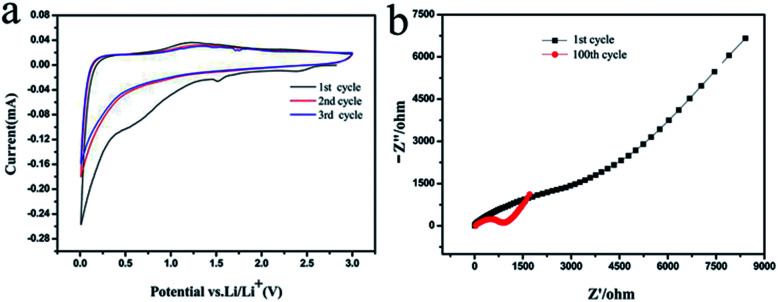
(a) Cyclic Voltammogram (CV) profiles of the CSC-2.5 sample and (b) the impedance curves of the 1st and 100th cycle of the CSC-2.5 sample.

In order to further characterize the surface structure changes during charging and discharging, we took the CSC-2.5 sample as an example to analyze its Cyclic Voltammogram (CV) and impedance curves. The Cyclic Voltammogram (CV) profiles in Fig. 8a displayed two reduction peaks around 0.65 V and 1.55 V in the first circle. The reduction peak at 0.65 V corresponded to the decomposition of electrolyte and the formation of solid electrolyte interphase (SEI) film.^28^ The reduction peak around 1.55 V was attributed to the complex irreversible reaction of lithium ions.^21^ The two reduction peaks disappeared in the subsequent cycles and no oxidation peak corresponded to the two reduction peaks, which demonstrated that a stable SEI film was formed in the first cycle. Therefore, the irreversible capacity of the first cycle was mainly caused by the SEI film in discharge process.^29^ In addition, the “hump” between 1.0–1.3 V was mainly due to the insertion and extraction of lithium ions in porous structure.^30^

The impedance curves of the 1st and 100th cycle of the CSC-2.5 sample were shown in Fig. 8b. During the first cycle, the high-frequency region had a larger semicircle because the formed SEI film increased the charge transfer resistance. After the 100th cycle, the semicircle in the high frequency region was significantly reduced, indicating that the charge transfer resistance was significantly reduced.^31^ The intercalation and deintercalation of lithium ions after many cycles may cause the thin walls inside the carbon material to be broken, and some channels and gaps were opened, so that the space where charge and lithium ions move freely increased, and the charge transfer resistance became smaller. This was consistent with the results described in Fig. 7a: during the 60th to 100th cycle, the intercalation and deintercalation of lithium ions resulted in a decrease in the volumetric strain energy and an increase in charge and discharge capacity.^32^ The impedance analysis before and after cycling support that the Li^+^ ions movement depended on the optimized pore and channel structure in biomass carbon.

## 4. Conclusions

A facile and effective method was developed to synthesize porous biomass carbon using corn stalk by carbonization and activation with CaCl_2_. After analyzing the formation mechanism, we found that CaCl_2_ was an important factor in the formation of porous structure. Porous structure can offer more active sites and shorten the transport paths for Li ions insertion/extraction. When the weight ratio of corn stalk powder and CaCl_2_ was 1 : 2.5, the CSC-2.5 sample had the largest specific surface area and electrochemical performance. The reversible capacity of the CSC-2.5 sample was 783.8 mA h g^−1^ after 100 cycles at 0.2C. Furthermore, after 60 cycle at various rates from 0.2C to 5C, the discharge capacity of the electrode can be restored quickly. All of these illustrated that our method could effectively synthesize the porous biomass carbon with excellent cyclic stability and superior rate capacity, which makes it one of the ideal candidates for biomass-derived the lithium ion batteries.

## Conflicts of interest

There are no conflicts to declare.

## Supplementary Material
